# Orodispersible Membranes from a Modified Coaxial Electrospinning for Fast Dissolution of Diclofenac Sodium

**DOI:** 10.3390/membranes11110802

**Published:** 2021-10-21

**Authors:** Tingbao Ning, Yangjian Zhou, Haixia Xu, Shiri Guo, Ke Wang, Deng-Guang Yu

**Affiliations:** 1 School of Materials Science and Engineering, University of Shanghai for Science and Technology, Shanghai 200093, China; 201850155@st.usst.edu.cn (T.N.); 192432632@st.usst.edu.cn (Y.Z.); 193742716@st.usst.edu.cn (H.X.); 1935023610@st.usst.edu.cn (S.G.); kora2009@163.com (K.W.); 2 Shanghai Engineering Technology Research Center for High-Performance Medical Device Materials, Shanghai 200093, China

**Keywords:** orodispersible membranes, coaxial electrospinning, poorly water-soluble drug, core-sheath nanofibers

## Abstract

The dissolution of poorly water-soluble drugs has been a longstanding and important issue in pharmaceutics during the past several decades. Nanotechnologies and their products have been broadly investigated for providing novel strategies for resolving this problem. In the present study, a new orodispersible membrane (OM) comprising electrospun nanofibers is developed for the fast dissolution of diclofenac sodium (DS). A modified coaxial electrospinning was implemented for the preparation of membranes, during which an unspinnable solution of sucralose was explored as the sheath working fluid for smoothing the working processes and also adjusting the taste of membranes. SEM and TEM images demonstrated that the OMs were composed of linear nanofibers with core-sheath inner structures. XRD and ATR-FTIR results suggested that DS presented in the OMs in an amorphous state due to the fine compatibility between DS and PVP. In vitro dissolution measurements and simulated artificial tongue experiments verified that the OMs were able to release the loaded DS in a pulsatile manner. The present protocols pave the way for the fast dissolution and fast action of a series of poorly water-soluble active ingredients that are suitable for oral administration.

## 1. Introduction

The effective delivery of poorly water-soluble drugs has been a longstanding and important issue in pharmaceutics due to their poor solubility in water [1,2,3]. During the past several decades, a wide variety of efforts have been paid to potential solutions, including both from new pharmaceutical excipients [4,5,6] and new methods for converting them in suitable dosage forms [7,8,9]. In this nano era, nanotechnologies and their products are playing an increasingly important role in providing novel strategies for resolving this problem [10,11]. One example is electrospinning and electrospun nanofibers [12,13,14]. 

**Figure 1:**
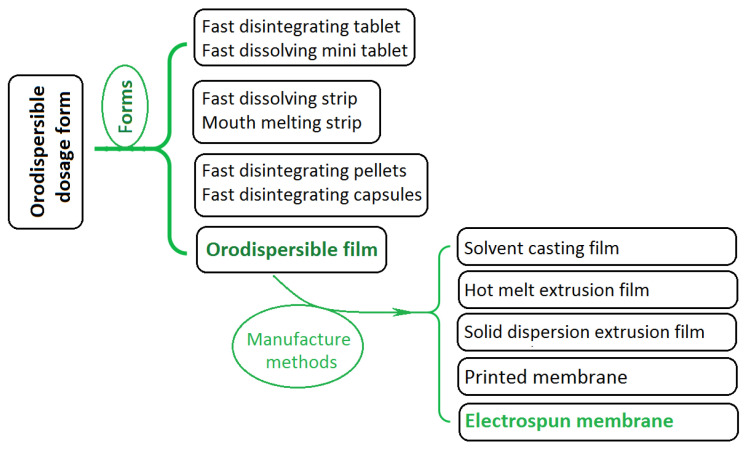
The progress and divisions of orodispersible dosage forms.

In pharmaceutics, orodispersible dosage is a popular form that provides convenience and has good compliance among patients in all disease conditions. Shown in Figure 1 is a diagram about the progress and division of orodispersible dosage during the past several decades. From a standpoint of “forms”, orodispersible dosage forms have the form of tablets (such as fast disintegrating tablets [15,16,17,18,19] and fast dissolving mini tablets [20,21,22], strips (such as fast dissolving strip [23,24] and mouth melting strip [25]), fast disintegrating pellets [26] and capsules (often with fast disintegrating particles in them) [27], and many new types of films or membranes [28,29,30,31]. Based on the manufacture methods, orodispersible membranes (OMs) can be further divided into several categories, such as the most common one-solvent casting film [32,33], hot melt extrusion film [34,35], solid dispersion extrusion film [36,37], printed membrane [38,39,40,41], and the present hot topic: electrospun nanofiber membrane [42,43,44].

Electrospinning, an abbreviation of electrostatic spinning, is an electrohydrodynamic atomization (EHDA) method [45,46,47]. These methods, including also electrospraying and e-jet printing, taking advantage of the facile interactions between the liquids and electrostatic energy for removing the solvents from the fluids rapidly to prepare solid products [48,49,50]. Often, the homogeneous distributions of solutes in the fluids can be propagated into the solid nanofibers or ultra-thin particles [51,52,53]. Thus, both electrospinning and electrospraying are frequently utilized in pharmaceutics for creating amorphous solid dispersions of poorly water-soluble drugs [54,55,56,57]. 

As for electrospun nanofiber membranes, their well-known properties such as small diameters of nanofibers, big porosity, and large surface areas have made them a powerful tool for resolving the dissolution issue of poorly water-soluble drugs [58]. Furthermore, based on the applications of soluble polymers as a filament-forming matrix, electrospun homogeneous nanofiber-based drug-polymer composites are frequently exploited to provide immediate or pulsatile release of poorly water-soluble drugs [59,60]. Thus, a wide variety of pharmaceutical excipients are explored to prepare fast dissolving membranes of poorly water-soluble drugs such as polyvinylpyrrolidone (PVP), poly(ethylene oxide), polyvinyl alcohol, pullulan, gelatin, and also beta-cyclodextrin [61,62,63,64,65]. However, applications of electrospun complicated nanostructures have received little attention. During the past several years, electrospinning is moving forward from the traditional single-fluid blending process [66] to coaxial [67,68], side-by-side [69,70], tri-axial [71,72], and other multiple-fluid processes [73,74] for generating core-sheath, Janus, tri-layer core-sheath and multiple-chamber nanostructures. These structures, on the one hand, should have advantages over those homogeneous drug-polymer nanocomposites, which have been seldom demonstrated in promoting fast dissolution of poorly water-soluble drugs, particularly their orodispersible membranes. On the other hand, these structures can be easily prepared from the unspinnable materials because often only one of the multiple fluids must be electrospinnable during a multi-fluid electrospinning process [68,69,70,71,72,73,74]. 

In this study, a new orodispersible membrane (OM) comprising electrospun core-sheath nanofibers is developed for the fast dissolution of diclofenac sodium (DS). A modified coaxial electrospinning was carried out to create the nanofiber membranes, during which an unspinnable solution of sucralose and PVP K10 was explored as the sheath working fluid for smoothing the working processes and also adjusting the taste of membranes. The core solution composed of DS and PVP K60 was spinnable. Their morphologies, physical state of components, and functional performances are compared with OMs from the traditional blending electrospinning processes.

## 2. Experimental

### 2.1. Materials

The drug DS (purity of 99.8%) was purchased from Shanghai Hua-Shi Great Pharmacy (Shanghai, China). PVP K60 (Mw = 360,000) and PVP K10 (Mw = 8000) were bought from Shanghai Aldrich Co., Ltd. (Shanghai, China). Anhydrous ethanol and phosphate buffer solution (PBS, 0.1 M, pH = 7.0) were obtained from the First Hangzhou Reagent Factory (Hangzhou, China). Other chemicals were analytical grade and water was double distilled before use. 

### 2.2. EHDA Processes

The apparatus for implementing all the EHDA processes was homemade. Two solutions were prepared and were guided to the spinneret as the sheath and core working fluids. The sheath fluid consisted of 5.0 g sucralose and 10.0 g PVP K10 in 100 mL anhydrous ethanol, and the core fluid was composed of 5.0 g DS and 8.0 g PVP K60 in 100 mL anhydrous ethanol. After some pre-experiments, the EHDA product collected distance was fixed at 20 cm. The environmental temperature and humidity were 21 ± 6 ℃ and 47 ± 4%, respectively. Other parameters such as applied voltage and fluid flow rates are included in Table 1. Three kinds of EHDA processes were conducted, and their products are denoted as E1 (a single-fluid electrospraying from the sheath fluid), E2 (a single-fluid electrospinning from the core fluid) and E3 (a modified coaxial electrospinning), respectively.

### 2.3. Characterizations

#### 2.3.1. Morphologies and Structures

Scanning electron microscopy (SEM) is exploited for assessing the surface morphologies of the three EHDA products and also the cross-sections of the core-sheath nanofibers E3. Before observations, the samples were pasted on the conductive adhesive tape and were sprayed a thin layer of Au for 90 s. A strip of nanofibers E3 was cut out and immersed into the liquid N_2_, and was manually broken after 20 min for preparing the samples of cross-sections.

#### 2.3.2. Physical State and Compatibility

X-ray diffraction (XRD, Bruker-AXS, Karlsruhe, Germany) and attenuated total reflectance-Fourier transform infrared spectroscopy (ATR-FTIR, Spectrum 100, Billerica, MA, USA) were carried out for evaluating the physical state and compatibility of components within the EHDA products. The ranges for 2θ in XRD and wavelength in FTIR were 0–60° (0.02°/s) and 4000 to 500 cm^−1^ (with a resolution of 1 cm^−1^). 

### 2.4. Fast Dissolution Performances 

The raw DS powders and EHDA products E2 and E3 were subjected to the following three sorts of fast dissolution measurements.

#### 2.4.1. Fast Wetting Process

Based on several papers and a Petri dish, an artificial tongue was developed for measuring the dissolution performances of the E2 and E3. 

#### 2.4.2. Drop Shape Analyses

The disappearance of a 3 μL double-distilled water was explored to assess the fast dissolution performances of E2 and E3 through a drop shape analysis instrument (DSA100, Kruss GmbH, Hamburg, Germany) 

#### 2.4.3. In Vitro Dissolution Tests

A UV spectrophotometer (Unico Instrument Co., Ltd., Shanghai, China) was exploited to quantitatively measure the concentration of DS in the solutions. DS has a maximum absorbance at λmax = 276 nm. Its standard equation of absorbance (A) to concentration (C, µg/mL) is A = 0.03265C-0.00134 (with a correlation coefficient of R = 0.9997 and within a linear range of 2~50 µg/mL). 

A Water Bath Constant-Temperature Shaker (SHZ-86, Jintan Shuibei Science Popularization Experimental Instrument Factory, Changzhou, China) holding seven conical flasks (containing 100 mL PBS, the added DS powders, E2 and E3 were 5.0, 13.0, and 20.5 mg, respectively) was utilized to implement the in vitro dissolution tests. The rotation rate of the shaker and the temperature were fixed at 50 rpm and 37 °C ± 0.5 ℃. At 1, 5, 10, 20, 30, 60 min, 5.0 mL of the dissolution media was drawn back for analyses and 5.0 mL fresh PBS was compensated. 

## 3. Results and Discussion

### 3.1. The EHDA Processes

**Figure 2:**
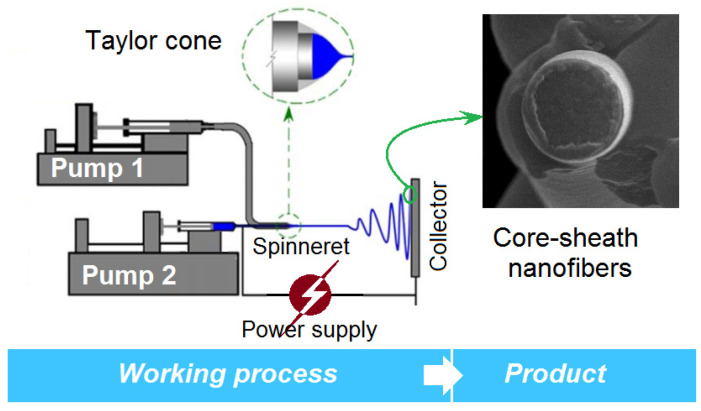
The modified coaxial electrospinning and the core-sheath nanostructure.

A diagram of the homemade EHDA apparatus is shown in Figure 2. As with any blending electrospinning apparatus or electrospraying system, it comprises four sections: one power supply, two pumps, a concentric spinneret and a collector. When both the sheath and core fluids were pumped to the spinneret, core-sheath nanofibers can be generated. However, when one of the core or sheath fluids is closed, the coaxial EHDA process will downgrade to a one-fluid EHDA process, and homogeneous EHDA products will be created from a single-fluid blending process. The properties of the single fluid will determine the products to be solid nanofibers or ultra-thin particles. 

**Figure 3:**
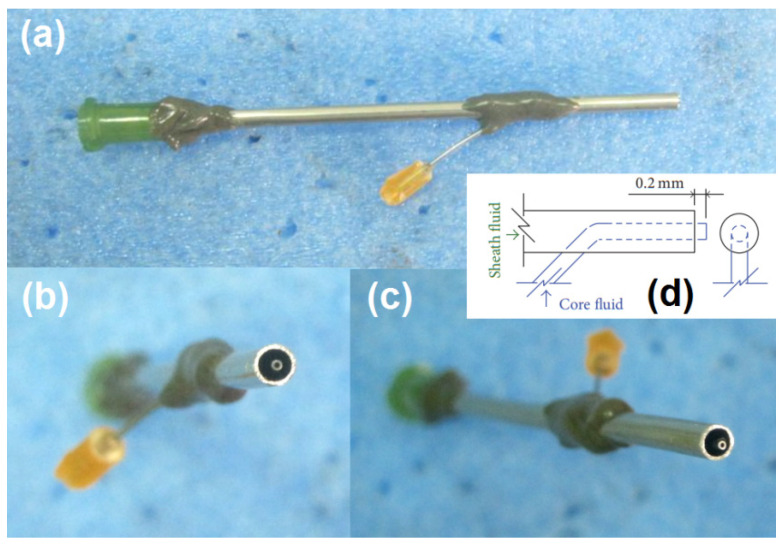
The concentric spinneret: (**a**) a full digital image; (**b**) the front image of the co-exist of double fluids; (**c**) the side view of the tip of the spinneret; (**d**) the quantitative design details of the spinneret.

Among the four sections within an EHDA apparatus, the spinneret is the most important. Often, the structures of spinneret determine the name of working processes (such as coaxial electrospinning due to a concentric spinneret [72,75]) and the final products (such as Janus nanofibers from an eccentric spinneret [70]). In the present study, the details about the homemade spinneret are exhibited in Figure 3. Figure 3a–c are digital images of the concentric spinneret from a full, a front, and a side view. The inner capillary and the outer capillary have a common axis, meaning it suitable for conducting a coaxial EHDA process. Meanwhile, the inner capillary is designed to be slightly projected out the outer capillary 0.2 mm. This design feature should benefit a the leading role of the core fluid during electrospinning and a prevention of mutual diffusion of the double working fluids. 

**Figure 4:**
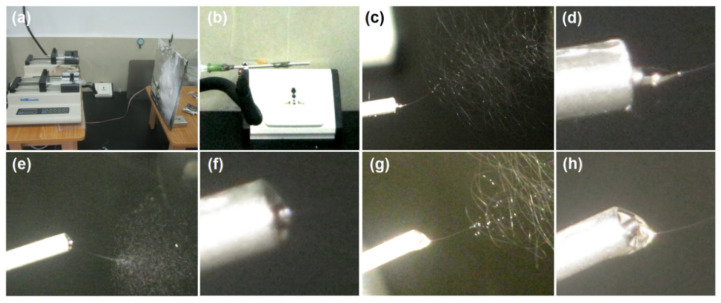
The EHDA processes: (**a**) a full digital image of the homemade electrospinning system; (**b**) an alligator clip is applied to transfer the high electrostatic energy to the working fluid; (**c**) and (**d**) a typical working process and Taylor cone for preparing E2, respectively; (**e**) and (**f**) a typical working process and Taylor cone for preparing E1, respectively; (**g**) and (**h**) a typical working process and Taylor cone for preparing E3, respectively.

In the present study, three sorts of EHDA process were carried out to generate three kinds of EHDA product. Shown in Figure 4 are a series of digital images of the typical working processes and the organization of apparatus. Figure 4a is a full digital image of the homemade electrospinning system, two pumps simultaneously drove the sheath and core working fluids to the concentric spinneret. Meanwhile, the high voltage was applied to the working fluids through an alligator clip (Figure 4b). When the sheath fluid is switched off, a single-fluid blending electrospinning of the core spinnable liquid can be clearly observed in Figure 4c. Correspondingly, the Taylor cone is recorded in Figure 4d. The homogeneous DS-PVP K60 nanofibers E2 were successfully prepared using this process. In contrast, When the core fluid is turned off, a single-fluid blending electrospraying of the sheath unspinnable solution can be observed in Figure 4e, with a small Taylor cone in Figure 4f. The homogeneous sucralose-PVP K10 particles E1 were generated through this process. When both the core and sheath fluids are switched on, the typical coaxial electrospinning process is given in Figure 4g. Obviously, the well-known three steps of electrospinning can be discerned, i.e., Taylor cone (an enlarged image is given in Figure 4h), a straight fluid jet, and the gradually enlarged bending and whipping loops due to the electrostatic repulsion. 

### 3.2. Properties of the EHDA Products

**Figure 5:**
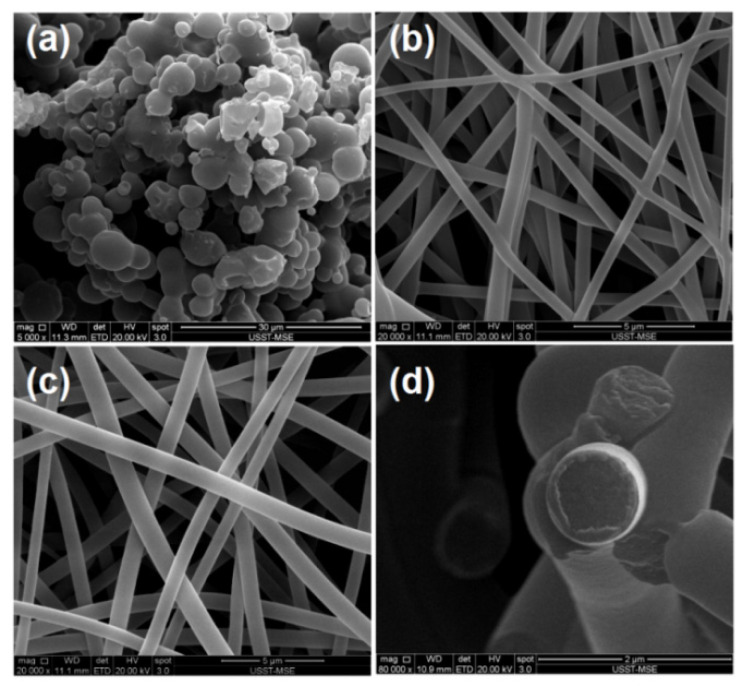
Scanning electron microscope (SEM) images of the prepared EHDA products: (**a**) E1; (**b**) E2; (**c**,**d**) surface and cross-section morphologies of E3, respectively.

The SEM images of the three sorts of EHDA product are shown in Figure 5. The particles E1 have an estimated diameter of 1.82 ± 0.34 μm. Some abnormal phenomena include satellites and clinging of particles can be found (Figure 5a). The nanofibers E2 from the blending electrospinning of core solution have a straight linear morphology and their estimated diameters are 0.64 ± 0.18 μm (Figure 5b). Similarly, the core-sheath nanofibers E3 from the modified coaxial electrospinning also exhibit linear morphology without any discernible beads-on-a-string or spindles-on-a-string phenomenon (Figure 5c). They have an average diameter of 0.81 ± 0.15 μm by estimation. 

The sheath unspinnable solutions have exerted some influences on the formation of core-sheath nanofibers E3. On the one hand, the added sheath fluid should make the nanofibers have a larger diameter due to more solutes passing through the nozzle in a unit time. On the other hand, the unspinnable sheath fluids should extend the drawing time of the core fluid under the electrostatic field due to an envelope effect. Thus, the cross-sections of nanofibers E3 were observed, which are exhibited in Figure 5d. The sheath sections have an estimated thickness of 0.12 μm, and the core diameter is about 0.57 μm, slightly smaller than the values of the homogeneous nanofibers E2. Meanwhile, the successful observation of core-sheath nanostructure has a close relationship with the different mechanical performances of sheath and core sections.

**Figure 6:**
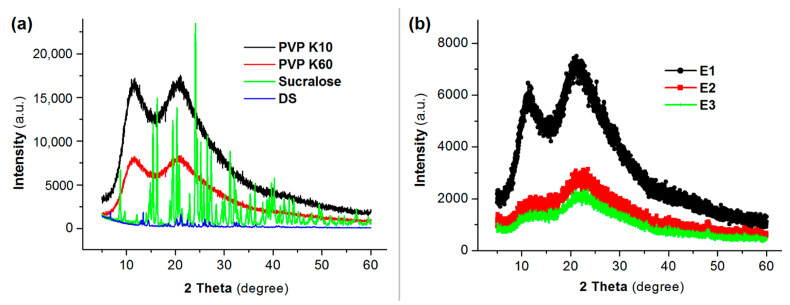
X-ray diffraction (XRD) patterns: (**a**) the raw materials DS, sucralose, PVP K60 and PVP K10; and (**b**) their EHDA products.

The XRD patterns of the raw materials (DS, sucralose, PVP K60 and PVP K10) and their EHDA products are included in Figure 6. Just as anticipated, both raw sucralose and DS powders have many sharp peaks in their XRD patterns, giving a hint that they are crystalline materials initially. In sharp contrast, both PVP K10 and PVP K60 have no any sharp peaks except two halos, suggesting they are amorphous polymers. After the EHDA processes, all the XRD patterns of the three types of EHDA product show no sharp peaks. These phenomena demonstrate that the EHDA products are completely amorphous composites, regardless of their formats of fibers or particles. During the EHDA processes, the solvents can be removed by the electrostatic energy at a time scale of several decades of milliseconds. The extremely fast drying processes would effectively convert the highly homogeneous distribution state of the solutes in the working fluids to the final solid EHDA products. Meanwhile, the solutes in the sheath fluids and the core fluids have little time to diffuse to each other to ensure the successful formation of core-sheath structures. 

**Figure 7:**
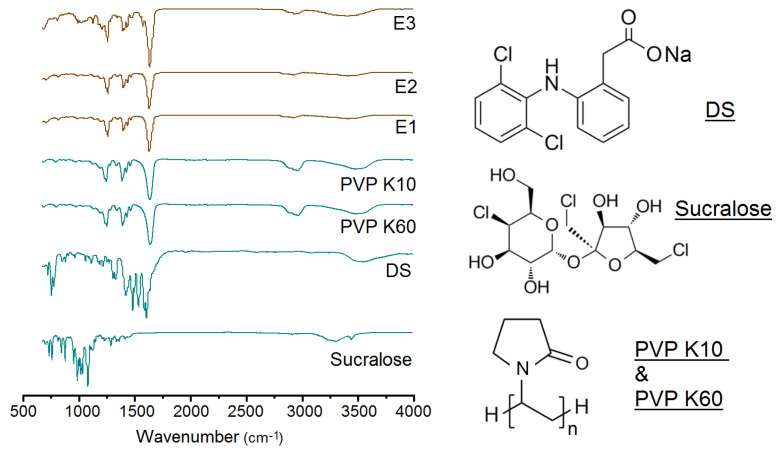
Attenuated total reflectance-Fourier transform infrared spectroscopy (ATR-FTIR) spectra of the raw materials (DS, sucralose, PVP K60 and PVP K10) and their EHDA products, and their molecular formula.

Shown in Figure 7 are ATR-FTIR spectra of the raw materials (DS, sucralose, PVP K60 and PVP K10) and their EHDA products, and their molecular formula. Both DS and sucralose have many sharp peaks in their finger region. However, when they were encapsulated in the EHDA products with PVP K10 and PVP K60, these sharp peaks almost completely disappeared. The reasons should be that DS and sucralose molecules have formed secondary interactions with the polymeric carriers. These secondary interactions include hydrogen bonding (such as protons in DS and sucralose molecules as donors and –C=O in PVP molecules as acceptor), hydrophobic interactions between the benzene rings of DS and long carbon chains of PVP, and also electrostatic interactions. These secondary interactions should be fine for making the EHDA products stabler for shipping and storage. 

### 3.3. Functional Performance of the EHDA Products

**Figure 8:**
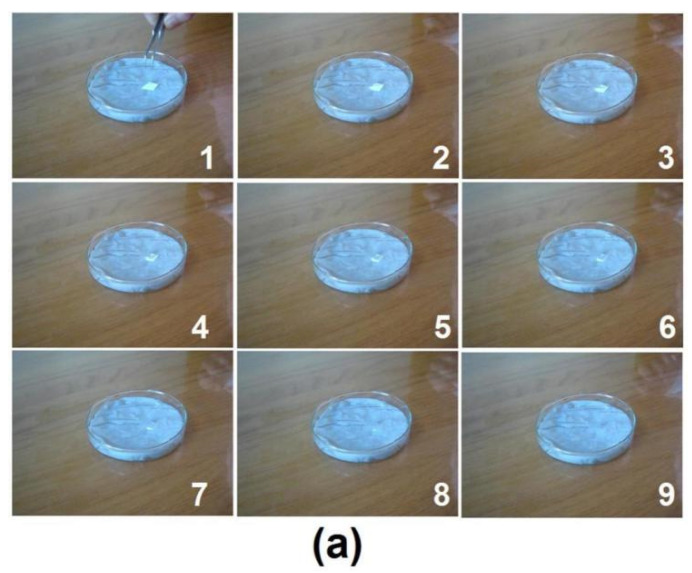

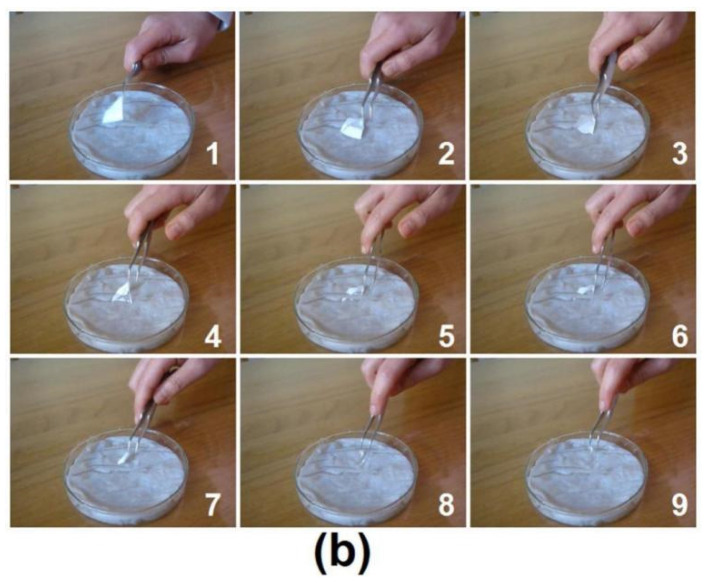
Fast disintegrating processes of E3 (**a**) and E2 (**b**).

To mimic the tongue, several papers were placed into a Petri dish, and small volume of water was utilized to wet out them. Then a small patch of EHDA products E3 was put onto the wet paper. The fast disintegrating process was captured by a camera, which is shown in Figure 8a. From “1” to “9”, the whole process cost only 15.8 ± 3.4 s (*n* = 3). Similarly, when a small patch of EHDA products E2 was placed onto the wet paper, it rapidly disappeared. The whole process from “1” to “9” in Figure 8b was only 16.1 ± 2.5 s (*n* = 3). There were no significant differences between their disintegrating processes. The added sheath sucralose showed no negative influences on the fast disintegrating of electrospun medicated membranes. 

**Figure 9:**
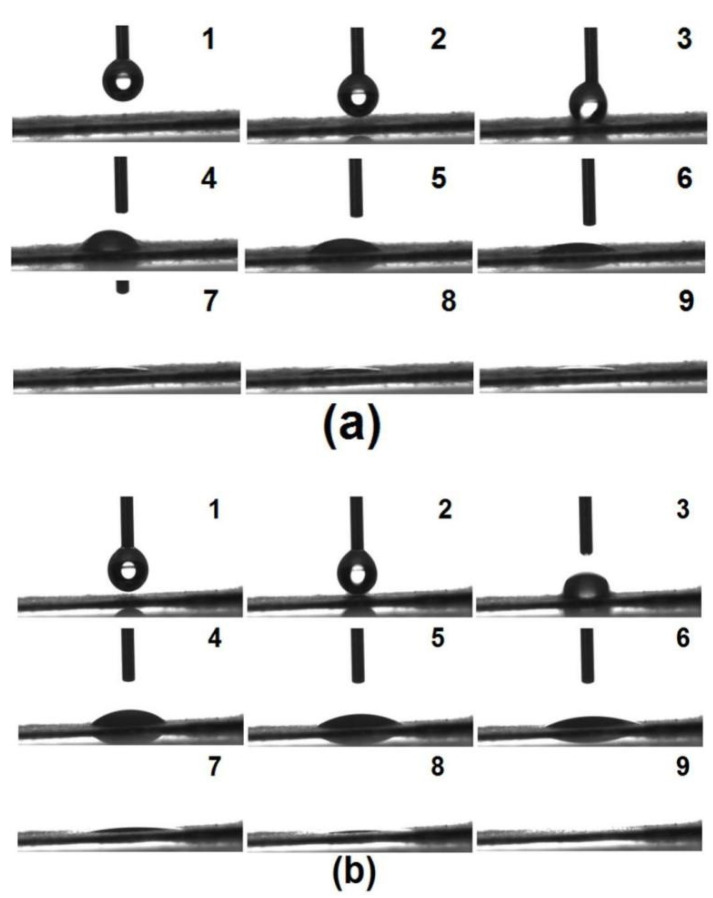
Tests on the water spreading processes of E2 (**a**) and E3 (**b**).

To further investigate the dispersible property of E2 and E3, a drop of water with a volume of 3 μL was added to the membranes. The recorded water spreading processes using the drop shape analysis instrument are included in Figure 9. In Figure 9a, the time from “1” to “9” was 11.1 ± 1.4 s (*n* = 3) for the water droplets disappeared in the EHDA E2. For the EHDA E2, the disappearance time of water droplets was 11.4 ± 1.2 s (*n* = 3), as shown from “1” to “9” in Figure 9b. 

**Figure 10:**
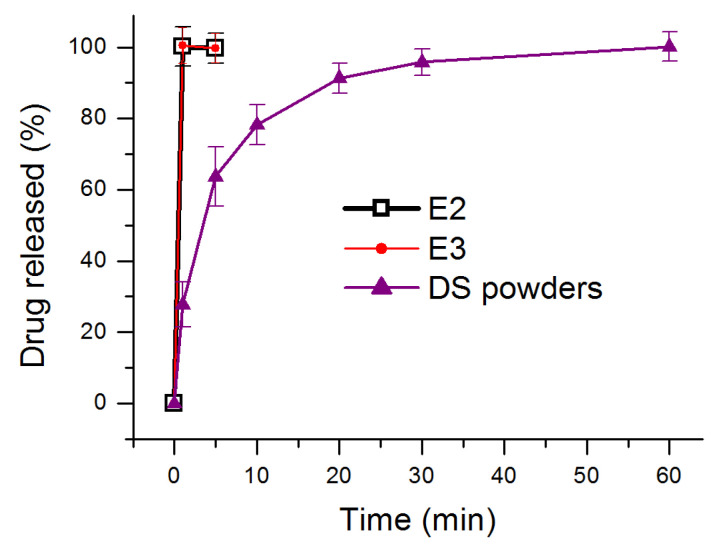
In vitro dissolution results of E2, E3 and DS powders.

Electrospinning is essentially a physical drying process, thus, the drug concentration in the EHDA products for E1, E2 and E3 were 0, 38.5% and 24.4%, respectively, which can be calculated according to the preparation conditions. The in vitro dissolution tests’ results are shown in Figure 10. Both EHDA E2 and E3 were able to free the loaded DS within 1 min. This is mainly for the following reasons: (1) hydrophilic matrices PVP K10 and PVP K60; (2) the properties of the electrospun membranes, such as small diameter of fibers and huge porosity; (3) the amorphous physical state of DS in the EHDA products. As for the DS powders, which have a white color with a size smaller than 0.75 mm, almost 1 h was needed to be fully dissolved. Manipulating a suitable drug release rate is always an important task in pharmaceutics [75,76,77,78]. The present protocols show a way for many similar drugs that can be administered orally.

### 3.4. Strategy for Developing Medicated Membrane Using Modified Coaxial Electrospinning 

DS is partially soluble in water, whose saturated solubility in PBS (pH = 7.0, 25 °C) is 1.13% [79]. As a popular non-steroidal anti-inflammatory drug, DS is available in a variety of dosage forms, particularly, there are many dosage forms for oral administration [80,81]. However, there are almost no reports about the OM of DS although OM is popular among patients. The reasons should be that DS is very bitter and the traditional orodispersible tablets may have a strong sense of gravel. Apparently, the present reported OMs comprising the electrospun core-sheath nanofiber membranes are able to eliminate the bitter taste by the sheath sucralose, and the amorphous physical state within the nanofibers should completely reject the sense of gravel for the patients. 

**Figure 11:**
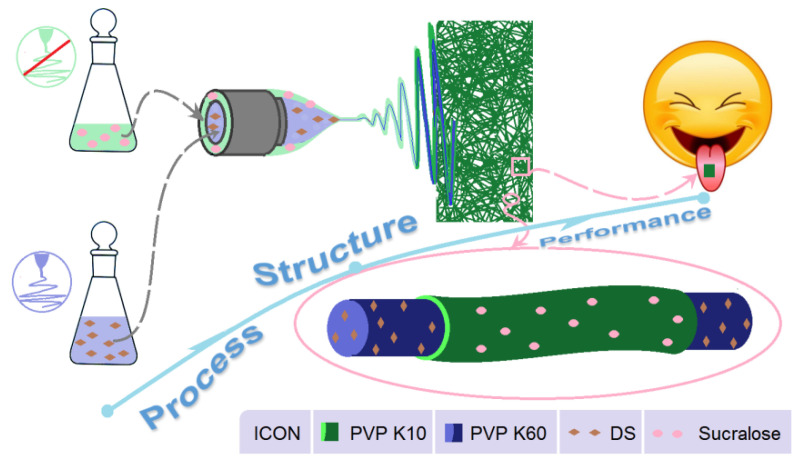
A process–structure–performance strategy for developing orodispersible membrane through the modified coaxial electrospinning.

Thus, the present report is not only a successful case study of OM for the drug DS, but also a new strategy for developing novel medicated membranes. A schematic about the strategy for developing OM through the modified coaxial electrospinning is exhibited in Figure 11. Clearly, it shows a process–structure–performance relationship. The advantage of the modified coaxial electrospinning over the traditional coaxial process is that all the materials (regardless of their electrospinnability) can be explored to create the sheath sections of core-sheath nanofibers, greatly expanding the capability of electrospinning in producing novel nanostructures. Certainly, based on the core-sheath nanostructures, a wide variety of drugs may be delivered through their OM dosage forms when fast actions are needed to relieve pain or bring down a fever. Besides drug delivery, the protocols reported here should be also useful for delivering nutrition in food science and engineering and for cosmetic applications [82,83,84].

## 4. Conclusions

In the present study, modified coaxial electrospinning was implemented to prepare a new type of core-sheath nanostructures in which the core drug–polymer composites were encapsulated by the sheath sucralose-polymer composites. Although the sheath fluid composed of sucralose and PVP K10 had no electrospinnability, the core-sheath nanofibers showed linear morphology with an average diameter of 0.81 ± 0.15 μm. XRD and ATR-FTIR results demonstrated that the drug DS presented in the EHDA products in an amorphous state due to its fine compatibility with the polymeric carrier. The artificial tongue experiments and drop shape analyses demonstrated that the prepared OMs from the single-fluid blending process and the coaxial process had high dispersible properties. In vitro dissolution tests showed that the OMs were able to release the loaded DS within 1 min, whereas the DS powders needed 1 h. Thus, the electrospun core-sheath nanofibers are good candidates for delivering DS through OMs thanks to fast disintegration of the drug and also the taste masking using sucralose. The protocols reported here should be useful for many other poorly water-soluble drugs, fast action of which for a better therapeutic effect is desired.

## Figures and Tables

**Table 1:** Process parameters for conducting three kinds of EHDA process.

No.	Process	Applied Voltage (kV)	Pumping Rate (mL/h)		Morphology
			Sheath ^a^	Core ^b^	
E1	1-fluid electrospraying	15	0.5	--	Microparticles
E2	1-fluid electrospinning	15	--	1.0	Nanofibers
E3	Coaxial electrospinning	15	0.5	1.0	Nanofibers

^a^ A ratio of 5% (*w*/*v*) sucralose and 10% (*w*/*v*) PVP K10 were dissolved into ethanol. ^b^ A ratio of 5% (*w*/*v*) DS and 8% (*w*/*v*) PVP K60 were dissolved into ethanol.
