# Correction: Ning et al. Orodispersible Membranes from a Modified Coaxial Electrospinning for Fast Dissolution of Diclofenac Sodium. *Membranes* 2021, *11*, 802

**DOI:** 10.3390/membranes16030096

**Published:** 2026-03-03

**Authors:** Tingbao Ning, Yangjian Zhou, Haixia Xu, Shiri Guo, Ke Wang, Deng-Guang Yu

**Affiliations:** 1 School of Materials Science and Engineering, University of Shanghai for Science and Technology, Shanghai 200093, China; 201850155@st.usst.edu.cn (T.N.); 192432632@st.usst.edu.cn (Y.Z.); 193742716@st.usst.edu.cn (H.X.); 1935023610@st.usst.edu.cn (S.G.); kora2009@163.com (K.W.); 2 Shanghai Engineering Technology Research Center for High-Performance Medical Device Materials, Shanghai 200093, China

In the original publication [1], no y-axis was included for the XRD patterns in Figure 6. The y-axis was originally omitted by the authors due to the qualitative nature of the analysis, different sampling for each specimen, and the scale ranges presenting potential difficulties in visualizing the data as one figure. However, for readers, the inclusion of a y-axis makes the XRD patterns easier to interpret and compare. Therefore, two coordinate systems are included as two separate subfigures to visualize the data, including y-axes. The correct Figure 6 is as below.

The authors state that the scientific conclusions are unaffected. This correction was approved by the Academic Editor. The original publication has also been updated.

## Figures and Tables

**Figure 6:**
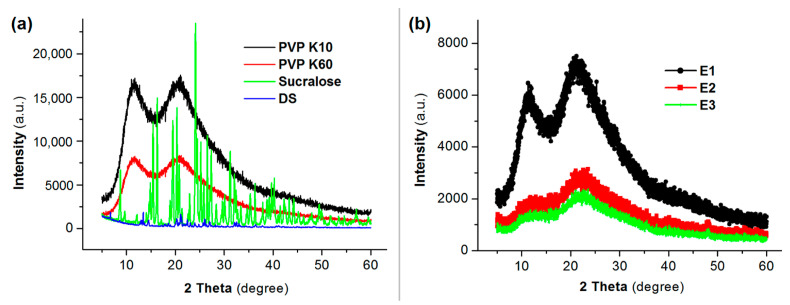
X-ray diffraction (XRD) patterns: (**a**) the raw materials DS, sucralose, PVP K60 and PVP K10; and (**b**) their EHDA products.
